# Host–microbial co-metabolites modulated by human milk oligosaccharides relate to reduced risk of respiratory tract infections

**DOI:** 10.3389/fnut.2022.935711

**Published:** 2022-08-04

**Authors:** François-Pierre Martin, Hanne L. P. Tytgat, Helle Krogh Pedersen, Deborah Moine, Aron C. Eklund, Bernard Berger, Norbert Sprenger

**Affiliations:** ^1^Nestlé Institute of Health Sciences, Nestlé Research, Société des Produits Nestlé S.A., Lausanne, Switzerland; ^2^Clinical Microbiomics, Copenhagen, Denmark; ^3^Nestlé Institute of Food Safety and Analytical Sciences, Nestlé Research, Société des Produits Nestlé S.A., Lausanne, Switzerland

**Keywords:** pediatric nutrition, milk oligosaccharides, gut microbiome, respiratory infection, metabolites

## Abstract

Human milk oligosaccharides (HMOs) are structurally diverse oligosaccharides present in breast milk, supporting the development of the gut microbiota and immune system. Previously, 2-HMO (2'fucosyllactose, lacto-*N*-neotetraose) compared to control formula feeding was associated with reduced risk of lower respiratory tract infections (LRTIs), in part linked to lower acetate and higher bifidobacteria proportions. Here, our objective was to gain further insight into additional molecular pathways linking the 2-HMO formula feeding and LRTI mitigation. From the same trial, we measured the microbiota composition and 743 known biochemical species in infant stool at 3 months of age using shotgun metagenomic sequencing and untargeted mass spectrometry metabolomics. We used multivariate analysis to identify biochemicals associated to 2-HMO formula feeding and LRTI and integrated those findings with the microbiota compositional data. Three molecular pathways stood out: increased gamma-glutamylation and *N*-acetylation of amino acids and decreased inflammatory signaling lipids. Integration of stool metagenomic data revealed some *Bifidobacterium* and *Bacteroides* species to be implicated. These findings deepen our understanding of the infant gut/microbiome co-metabolism in early life and provide evidence for how such metabolic changes may influence immune competence at distant mucosal sites such as the airways.

## Introduction

Human milk is considered the best food to nourish a growing infant. The World Health Organization, therefore, recommends exclusive breastfeeding until 6 months of age and continued breastfeeding after the introduction of complementary foods up to 2 years of age or longer (1). Human milk is a rich biological fluid adapted by evolution to provide nutrients to the growing infants, but also to support an age-appropriate development of their microbiome and immune system. Human milk is rich in a plethora of structurally diverse oligosaccharides, collectively known as human milk oligosaccharides (HMOs). They form the third largest solid component of human milk and come in a large structural variety based on the combination of their monosaccharide subunits: fucose, *N*-acetyl-neuraminic acid, *N*-acetyl-glucosamine, galactose, and glucose (2).

HMOs are known to support the development and maintenance of a healthy microbiota and immune system, two systems that mature in tandem in early life. HMOs steer microbiome development by influencing its composition *via*, e.g., bifidogenic (3–5) and anti-pathogenic effects (6), and may also directly interact with the gut epithelium, modulating physical microbe–host interactions (7). Age-appropriate development and maturation of the microbiome have been shown to be crucial for lifelong health through the modulation of immune competence (8–11). Many factors such as C-section birth, antibiotic use, and a lack of, or limited period of, breastfeeding have a negative impact on the development of the microbiome. Such changes to the developing microbial-immune handshake are paralleled with a higher infection risk in early life and increased susceptibility to allergic inflammation later in life (12–16).

Given the role of HMOs in microbiome and immune development, efforts to provide HMO-mediated health benefits to formula fed or partially breastfed infants are of particular interest. Previously, we have reported on the safety of an infant formula supplemented with two HMOs: 2'fucosyllactose (2'FL) and lacto-*N*-neotetraose (LNnT) (17). Both, 2'FL and LNnT positively regulate infants' bifidobacteria abundance and metabolic activity, known to support a healthy imprinting of the developing immune system (18, 19).

The 2'FL and LNnT-supplemented (2-HMO) formula was well-tolerated, supported age-appropriate growth, and associated with a reduction in reported incidence of lower respiratory tract illnesses (LRTIs) and medication use (antipyretics and antibiotics) compared to a control group receiving formula without HMOs (17). In particular, the lower risk of requiring antibiotics over the first year of life was coupled to an HMO-mediated shift in the infant fecal community types (FCTs) toward that observed in breastfed reference infants (20). A combination of machine learning models comparing positive and negative LRTI cases, together with *in vitro* experiments, highlighted a possible protective role of bifidobacteria, especially *Bifidobacterium longum* subsp. *infantis*, through their effect on the gut ecology (21).

We previously described that relative stool content of acetate, butyrate, 5-aminovalerate, succinate, and fucosyl-glycans were among the main identified metabolic features discriminating between cases and controls for respiratory illnesses using proton nuclear magnetic resonance spectroscopy (^1^H NMR) metabolomics. In addition, we reported that higher relative acetate compared to other short-chain fatty acids, likely combined with additional *Bifidobacterium* species-driven metabolites, may explain the observed clinical outcomes of reduced risk for bronchitis and LRTI in infants fed a 2-HMO formula (21).

In this study, we aimed to deepen our understanding of the molecular processes explaining the observed lower incidence of LRTIs in infants receiving the 2-HMO formula. We further studied the impact of HMO metabolization on stool biochemical composition by integrating novel data from a sensitive mass spectrometry (MS)-based metabolic profiling approach with novel metagenomic analyses of the samples. We subsequently discuss how insights generated in this combined data set might contribute to a healthy immune system imprinting from the gut to the airways and to a reduction of the risk of LRTIs in early life.

## Methods

### Clinical trial

A randomized, controlled, multicenter clinical trial was designed with the primary objective to evaluate non-inferiority of weight gain from enrollment to 4 months of age, comparing infants fed a control and a 2-HMO formula. The 2-HMO formula consisted of control formula (protein cow's milk protein-based) in which 1.5 g/L lactose was replaced by a 2:1 mixture of two HMOs, respectively, 2′FL and LNnT at 1.5 g/L. Further details of the trial, including randomization and blinding procedures, are outlined in Puccio et al. (17). Secondary objectives included the evaluation of differences between the formula groups in body weight, body length, head circumference, digestive tolerance, formula compliance, stool protein markers for intestinal status, stool microbiota, medication use, and morbidity through 12 months of age. Infection-related morbidity was assessed based on an *a priori* formulated hypothesis that the two HMOs act preventive (20).

In this study, we focused on a random subset (*n* = 80) of the per-protocol formula-fed population as defined in Berger et al. (20) for whom we had sufficient stool samples left for next-generation sequencing and MS-based untargeted metabolite profiling. In particular, 42 infants receiving control formula were included, and 38 of the 2-HMO formula group (Supplementary Figure 1). Adverse events were reported over the course of the first year of life of participating infants. More details of the trial are available in Supplementary material and the aforementioned papers (17, 20). The trial is registered at www.clinicaltrials.gov with the number NCT01715246.

### Analysis of stool microbiota composition

Total DNA was extracted from frozen fecal matter using the QIAamp DNA stool Mini Kit (QIAGEN) following the manufacturer's instructions apart from the addition of mechanical disruption steps (4 × 60s FastPrep in Lysing Matrix B tubes of MP Biochemicals) (22). DNA libraries were prepared using the Nextera XT protocol and Nextera DNA Sample Preparation Kit. Samples were sequenced on an Illumina HISeq instrument with PE 100 reads using six high-output flow cells. Taxonomic relative abundances were calculated using the metagenomic species (MGS) approach, which enables quantification of both known characterized and uncharacterized microbial species (23) (full details are outlined in the Supplementary material section).

Gut health markers (i.e., calprotectin, α1-antitrypsin, and elastase) were quantified as described earlier (cf. Supplementary materials) (21).

### Metabolomics of stool samples

Biochemical composition of stool samples was assessed using ^1^H NMR according to a well-established metabolomics protocol (21, 24, 25). To complement the biochemical analysis, MS-based untargeted metabolomics was conducted. Briefly, samples were, after short processing, divided into five fractions: two for analysis by two separate reverse phase ultrahigh performance liquid chromatography-tandem mass spectroscopy (RP/UPLC-MS/MS) methods using positive ion mode electrospray ionization (ESI), one for analysis by RP/UPLC-MS/MS using negative ion mode ESI, one for analysis by HILIC/UPLC-MS/MS using negative ion mode ESI, and one backup. Raw data were extracted, peak-identified, and QC processed using hardware and software from Metabolon Inc. (München, Germany). Full details of the analysis are available in Supplementary material.

### Statistics

Metabolomics data were subjected to Welch's two-sample *t*-tests and random forest analyses. For all analyses, missing values, if any, were imputed with the observed minimum for that compound. The statistical analyses were performed on natural log-transformed data. Additional multivariate data analysis was conducted using the software package SIMCA-P+ (version 16.0, Sartorius, Umeå, Sweden). Principal component analysis (PCA) was first employed to explore the variance within data set and assessment of major confounders (26). To identify metabolic differences that were statistically significant between groups, we employed partial least-squares regression analysis, and its modification, orthogonal projection to latent structures (OPLS) (27). Pre-filtering of the most influential variables was performed considering an OPLS coefficient above 0.22 and an OPLS variable influence on projection (VIP): threshold set at 1.5. Additionally, we included few metabolites that significantly differed between control and 2-HMO feeding groups in a univariate model and also stood out based on background knowledge of their importance, although they did not pass the defined threshold used here (e.g., DiHOMEs).

MGSs and metabolites that were detected in <10 of the 80 formula-fed infants were filtered out, and pairwise interdomain correlations between the normalized abundances of the remaining 124 MGSs and 50 metabolites were evaluated by Spearman's correlation (two-sided). The Benjamini–Hochberg procedure was used to control the false discovery rate (FDR) at 10% (i.e., concurrently on the entire matrix of all 6,200 pairwise combinations of metabolites and MGSs). MGSs and metabolites with at least one significant correlation (FDR ≤ 10%) were displayed in a heat map. The rows and columns of the heat map were ordered based on hierarchical clustering of Spearman's correlation coefficient (SCC) values using Euclidean distance as dissimilarity measure and Ward clustering.

Each of the 27 metabolites and 30 MGSs with at least one significant correlation in the procedure above were compared among (i) formula groups (control vs. 2-HMO formula) and (ii) LRTI groups (0 vs. ≥1 incidence of LRTI) with two-sided Mann–Whitney *U-*tests and Cliff's Delta as effect size using the wilcox.exact function from the *exactRankTests* R packages and the cliff.delta function from the *effsize* R package, respectively. The Benjamini–Hochberg procedure was used to control the FDR at 10% for each phenotype individually.

All analyses were run using the R software (v. 4.0.3).

### Study approval

The clinical trial was approved by the institutional review board(s) of the Dipartimento Materno Infantile AOUP “Paolo Giaccone,” Universita di Palermo, Palermo, Italy, and the Department of Pediatrics at Jessa Hospital in Hasselt, Belgium. The trial was conducted according to the Declaration of Helsinki and the International Conference on Harmonization guidelines for Good Clinical Practice. Parents or legal guardians of each infant provided informed consent before enrollment.

## Results

### Cohort description

A subset of stool samples of a randomized controlled trial, assessing the safety, efficacy, and effects of 2-HMO formula compared to test formula were further analyzed to explore potential relations between stool microbiota composition and functionalities, metabolites, stool biomarkers (inflammation, barrier function), and reported LRTIs. The full cohort and trial were described earlier (17, 20), and detailed information can be found in the “Materials and methods” section as well as a subject flowchart in Supplementary Figure 1. LRTI adverse events were defined using the preferred terms: bronchiolitis, bronchitis, pneumonia, LRTI, LRTI viral, respiratory syncytial virus (RSV) bronchiolitis, RSV bronchitis, respiratory infection viral. Any number of LRTI incidences, between 3 and 12 months of age, was counted as a case (Y) and no incidence as control (N). For some infants, several incidences of LRTI were reported (52, 22, 3, and 3 infants showing 0, 1, 2, and 3 events, respectively). In total, 3-month fecal samples of 28 LRTI-positive and 52 LRTI-negative infants were analyzed, which were distributed over both feeding groups (2-HMO vs. control formula) (Supplementary Figure 1; Table 1).

**Table 1:** Characteristics of infants (*n* = 80) included in this study.

**Infant characteristics**	**Control formula**	**2-HMO formula**	**Control formula with LRTIs**	**2-HMO formula with LRTIs**	**Control formula without LRTIs**	**2-HMO formula without LRTIs**
Sex (*n*, % male)	24 (57)	20 (52)	13 (72)	6 (60)	11 (46)	14 (50)
Gestational age (weeks)	39.4 ± 1	39.2 ± 1.1	39.5 ± 0.9	39.3 ± 1.1	39.3 ± 1	39.1 ± 1.1
Siblings (*n*, % yes)	25 (42)	23 (60)	12 (70)	7 (70)	13 (50)	16 (60)
C-section (*n*, % yes)	13 (31)	13 (34)	9 (50)	3 (30)	4 (16)	10 (36)
Weight at birth (kg)	3.4 ± 0.4	3.4 ± 0.4	3.5 ± 0.3	3.4 ± 0.3	3.3 ± 0.4	3.3 ± 0.5
Length at birth (cm)	50.2 ± 1.8	49.9 ± 1.8	50.4 ± 1.6	50.5 ± 1.9	50 ± 1.9	49.6 ± 1.7

*Data are shown as mean ± SD or n (%)*.

### Stool biochemical composition reveals several molecular pathways modulated by HMOs

**Figure 1:**
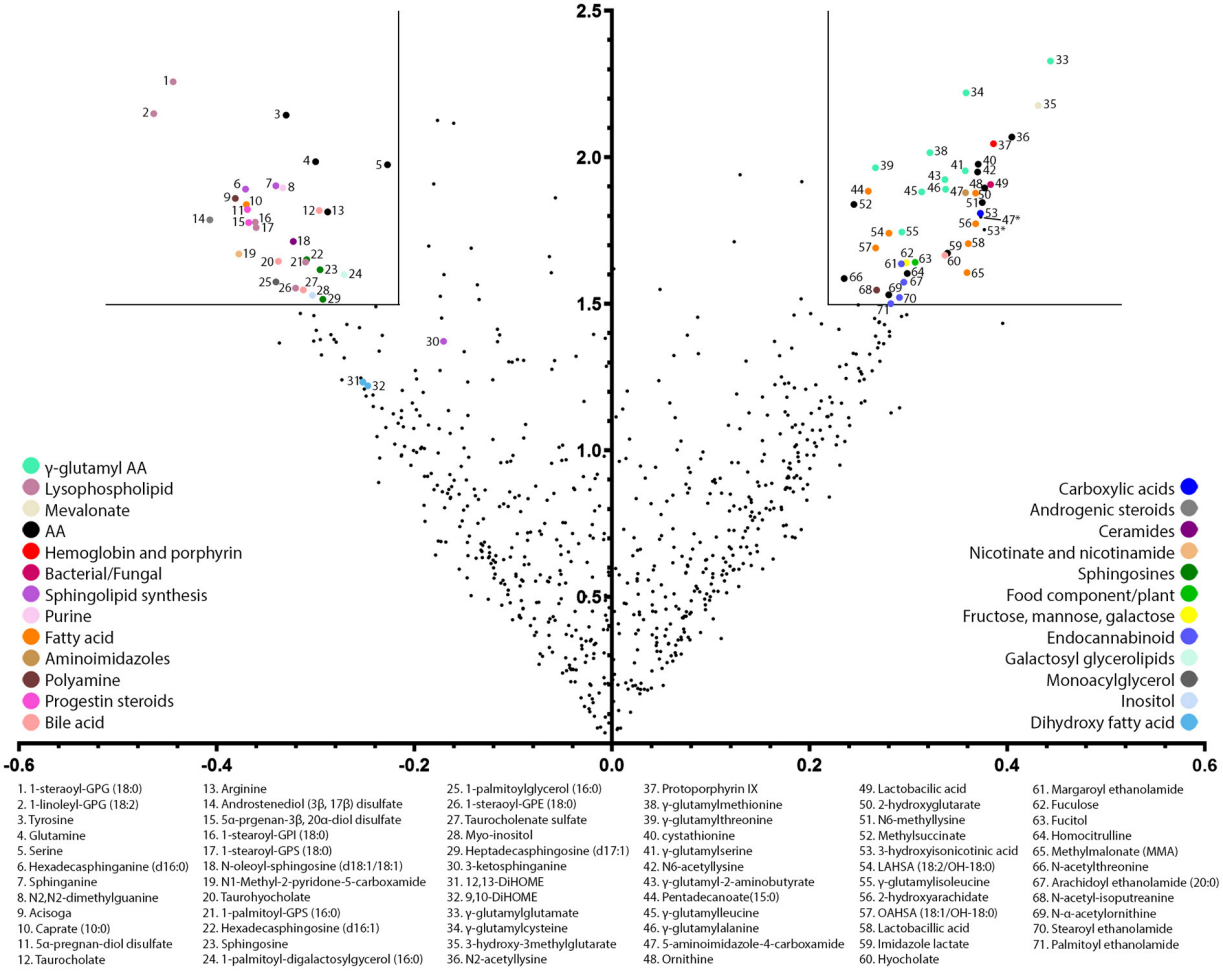
Volcano plot of most discriminating stool metabolites between infants receiving a 2-HMO and control formula. The volcano plot is a scatter plot of variables in which the x-axis corresponds to the OPLS correlation coefficient (pcorr) and the y-axis to the OPLS variable influence on projection (VIP). Pre-filtering of the most influential variables was performed considered an OPLS coefficient above 0.22 and an OPLS VIP: threshold set at 1.5. Notably, 73 selected variables are color-coded according to their major metabolic pathway, and their biochemical names are listed.

Here, we explored the metabolites present in stool of infants receiving 2-HMO formula vs. control formula further using a untargeted metabolomics approach based on liquid chromatography mass spectrometry methods. A total of 743 known biochemical species were detected and measured. Biochemical differences between the two feeding groups were modeled using an orthogonal projection to latent structures discriminant analysis (OPLS-DA, one predictive component, one orthogonal component, R^2^X value = 0.09, Q^2^Y value = 0.38). This resulted in 73 biochemical variables considered to be among the most discriminating metabolites in stool according to formula type, i.e., control vs. 2-HMO formula (Figure 1). In particular, the presence of HMOs in infant formula resulted in major changes in lipid and amino acid metabolism, most notably in phospholipid/sphingomyelin metabolism and the gamma-glutamylation and *N*-acetylation of amino acids (full list in Supplementary material 1).

The shift in amino acid metabolism induced by HMO metabolization was notably marked by increased concentrations in six *N*-acetylated-amino acids (NAAs) and nine gamma-glutamylated amino acids (GGAAs) (Table 2). Concomitantly, some of the free amino acid precursors tended to be reduced, namely, arginine, glutamine, serine, and tyrosine (Table 2).

**Table 2:** Overview of selected metabolites in stool content of infants receiving HMO formula compared to control formula, with fold of changes and associations to LRTIs.

**Molecular pathways**	**Metabolites**	**Effect of 2-HMO formula**				**Associations with LRTIs**	
		**Fold change**	***p*-value**	**OPLS Coefficient**	**OPLS VIP**	**OPLS Coefficient**	**OPLS VIP**
N-acetyl AA metabolism	N2-acetyllysine	2.32	0.001	0.40	2.07	−0.20	0.84
	N6-acetyllysine	2.43	0.0004	0.37	1.95	−0.32	1.30
	N-acetylserine	1.36	0.08	0.25	1.33	−0.21	1.00
	N-acetylthreonine	1.56	0.02	0.23	1.59	−0.19	0.83
	N-α-acetylornithine	1.49	0.02	0.28	1.53	−0.12	0.45
	N-δ-acetylornithine	1.82	0.07	0.27	1.39	−0.22	0.97
y-glutamyl AA (GGAAs) metabolism	y-glutamyl-2-aminobutyrate	**1.63**	**0.17**	**0.34**	**1.92**	**-0.47**	**1.91**
	y-glutamylalanine	1.50	0.07	0.34	1.89	−0.34	1.38
	y-glutamylcysteine	3.53	0.01	0.36	2.22	0.03	0.92
	y-glutamylglutamate	**1.51**	**0.05**	**0.44**	**2.33**	**-0.42**	**1.75**
	y-glutamylisoleucine	1.75	0.06	0.29	1.75	−0.34	1.41
	y-glutamylleucine	1.55	0.06	0.31	1.88	−0.34	1.46
	y-glutamylmethionine	**2.29**	**0.10**	**0.32**	**2.02**	**-0.37**	**1.53**
	y-glutamylserine	1.75	0.01	0.36	1.96	−0.12	0.62
	y-glutamylthreonine	**1.63**	**0.07**	**0.27**	**1.97**	**-0.41**	**1.67**
AA Metabolism	Arginine	0.71	0.10	−0.29	1.81	0.32	1.35
	Cystathionine	2.44	0.02	0.37	1.98	−0.14	0.84
	Glutamine	**0.73**	**0.02**	**-0.30**	**1.99**	**0.34**	**1.52**
	Homocitrulline	1.48	0.02	0.30	1.60	0.06	1.12
	imidazole lactate	1.59	0.002	0.34	1.67	−0.02	0.69
	Methylsuccinate	1.34	0.29	0.24	1.84	0.18	1.07
	N6-methyllysine	1.59	0.0003	0.37	1.85	0.05	0.54
	Ornithine	1.63	0.01	0.38	1.90	−0.06	0.36
	Serine	**0.78**	**0.01**	**-0.23**	**1.98**	**0.44**	**1.92**
	Tyrosine	**0.71**	**0.01**	**-0.33**	**2.14**	**0.43**	**1.76**
Sphingolipid synthesis	3-ketosphinganine	0.58	0.02	−0.17	1.37	0.51	1.98
	Hexadecasphinganine (d16:0)	0.71	0.003	−0.37	1.89	0.32	1.29
	Sphinganine	**0.65**	**0.003**	**-0.34**	**1.90**	**0.54**	**2.15**
Sphingosines	Heptadecasphingosine (d17:1)	0.70	0.01	−0.29	1.52	0.35	1.37
	Hexadecasphingosine (d16:1)	**0.69**	**0.02**	**-0.31**	**1.65**	**0.38**	**1.52**
	Sphingosine	**0.76**	**0.01**	**-0.30**	**1.62**	**0.50**	**2.00**
Dihydroxy fatty acids	12,13-DiHOME	0.88	0.04	−0.25	1.23	−0.04	0.38
	9,10-DiHOME	0.85	0.02	−0.25	1.22	−0.14	0.62

*The table lists all measured metabolites annotated to the N-acetyl AA metabolism, gamma-glutamyl AA (GGAAs) metabolism, AA metabolism, sphingolipid synthesis, sphingosines, and dihydroxy fatty acids pathways, a complete list is provided in Supplementary material 1. Fold change: difference between 2-HMO and control formula; OPLS coefficient: correlation coefficient between the metabolite and the feeding group (i.e., 2-HMO or control formula) or the LRTI incidence, derived from multivariate model; OPLS VIP: weight of the variable in the multivariate models. Variables are highlighted in bold when significance above OPLS coefficient threshold (0.22) and an OPLS VIP threshold (1.5) for both treatment and LRTI OPLS models*.

Infants fed the 2-HMO formula showed a distinct stool lipid signature, mainly pertaining to HMO-related remodeling of phospholipids and sphingomyelins. Changes in two dihydroxy fatty acids were also observed (12,13-DiHOME and 9,10-DiHOME). The changes in phospholipids and sphingomyelins are manifested by a reduction in six lysophospholipids and six sphingolipids (e.g., sphingosines and sphinganine metabolites). These metabolic changes were concomitant to an increase in seven free fatty acids and four endocannabinoid lipids (palmitoyl, arachidoyl, margaroyl, and stearoyl ethanolamides) (Table 2). Additional changes in four bile acids (hyocholic, taurocholic, taurocholenic, and taurohyocholic acids) may be related to changes in lipid digestion and absorption in the digestive tract (Supplementary material 1).

### HMO modulation of GGAAs and sphingolipids is associated with lower incidence of LRTI

Using a multivariate data analysis approach, we explored the associations between stool metabolites and LRTI incidence in all formula-fed infants, with emphasis on detecting whether HMO-modulated metabolites correlated with the observed lower incidence of LRTI between 3 and 12 months of age (17, 21). Biochemical differences in stool at 3 months of age were modeled using OPLS-DA between infants who experienced any LRTI or not up to 12 months of age (one predictive component, one orthogonal component, R^2^X value = 0.11, Q^2^Y value = 0.41). The contribution of different variables to the model is reported in Table 2 and fully listed in Supplementary material 1.

Among the HMO-modulated stool metabolites, GGAAs were found to be negatively associated to LRTIs, while sphingolipids and free amino acids correlated positively to later LRTIs. Despite lower statistical importance, NAAs tended to be negatively associated with LRTI (Table 2). Taken together, these observations indicate that HMOs drive changes in the gut functional ecology that are associated to the observed lower LRTIs.

### Integration of all omics data reveals *Bifidobacterium* and *Bacteroides* species as potential contributors to metabolic phenotypes linked to LRTI protection

**Figure 2:**
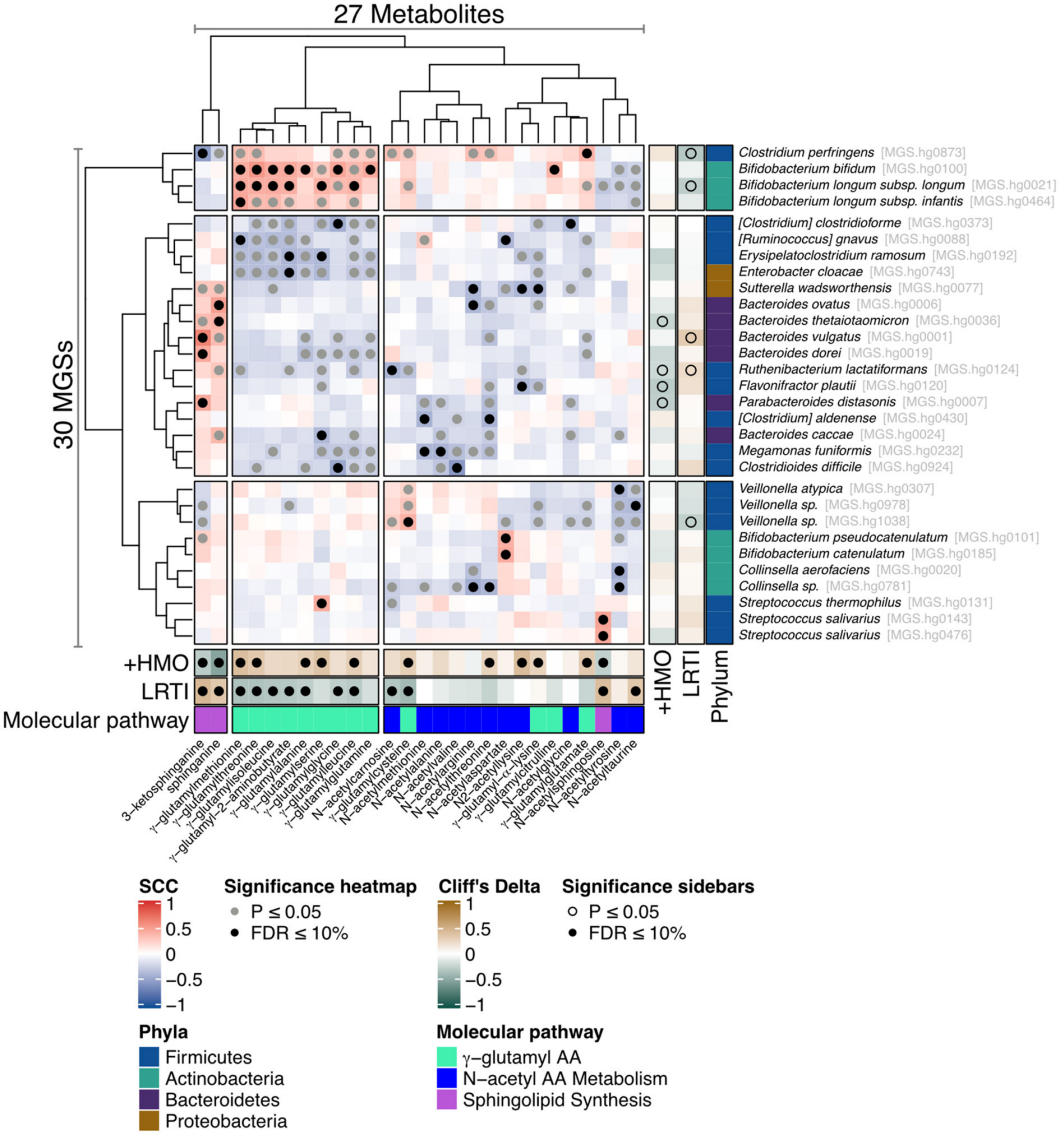
Heat map illustrating the correlation between metagenomic species (MGSs) and stool metabolites. Association map of the three-tiered analyses integrating the phenome, the gut microbiome, and the stool metabolome in the 80 formula-fed infants with available metabolomics data. The main “heat map” panel shows SCC between MGSs and the stool metabolites. Statistically significant correlations are indicated with filled gray (Spearman's correlation *p* ≤ 0.05) or black (Spearman's correlation FDR ≤ 0.1) circles. The right and bottom “sidebar” panels show associations between the same MGSs and metabolites, respectively, and feeding group (+HMO for the 2-HMO feeding group) or incidence of LRTI. The colors indicate the direction and magnitude of the association (Cliff's Delta), where brown means the MGS or metabolite is more abundant in 2-HMO-fed infants or in infants who experienced one or more episodes of LRTI, and green means the MGS or metabolite is more abundant in control-fed infants or in infants who did not experience an LRTI episode.

To define which bacterial species may contribute to the molecular pathways generating the metabolites associated to LRTI incidence and feeding group (2-HMO formula vs. control formula), the metabolomics data were integrated with a metagenomics analysis of the microbiome. The 50 considered metabolites were preselected because they belong to the three main pathways identified. They include 22 NAAs, 15 GGAAs, 2 dihydroxylated fatty acids (DiHOME), sphingosine, sphinganine, and 9 other lipids in the sphingolipid biosynthesis pathway. Several statistically significant associations were found between LRTI phenotype or feeding group with the three identified groups of key metabolites, namely, GGAAs, NAAs, and sphingolipids using univariate Mann–Whitney *U*-test (Figure 2; Supplementary material 2). Furthermore, the analysis revealed that some of these metabolites correlated with distinct bacterial species, thus indicating that these might harbor the related metabolic pathways. The analysis revealed that sphinganine and 3-keto-sphinganine were most strongly correlated with *Bacteroides* species, while GGAAs were most strongly correlated with *Bifidobacterium* species, namely, *B. bifidum, B. longum* subsp. *longum*, and *B. longum* subsp. *infantis* (Figure 2; Supplementary material 2). The associations between NAAs and the microbiome were more scattered, as noted by a multitude of bacterial species that might thus be equipped with enzymatic functions involved in the synthesis of NAA metabolites. Among them were also two bifidobacteria, *Bifidobacterium pseudocatenulatum* and *Bifidobacterium catenulatum* (Figure 2).

### Integration of stool metabolites and gut health markers further supports their contribution to reduced risk of LRTI in early life

**Figure 3:**
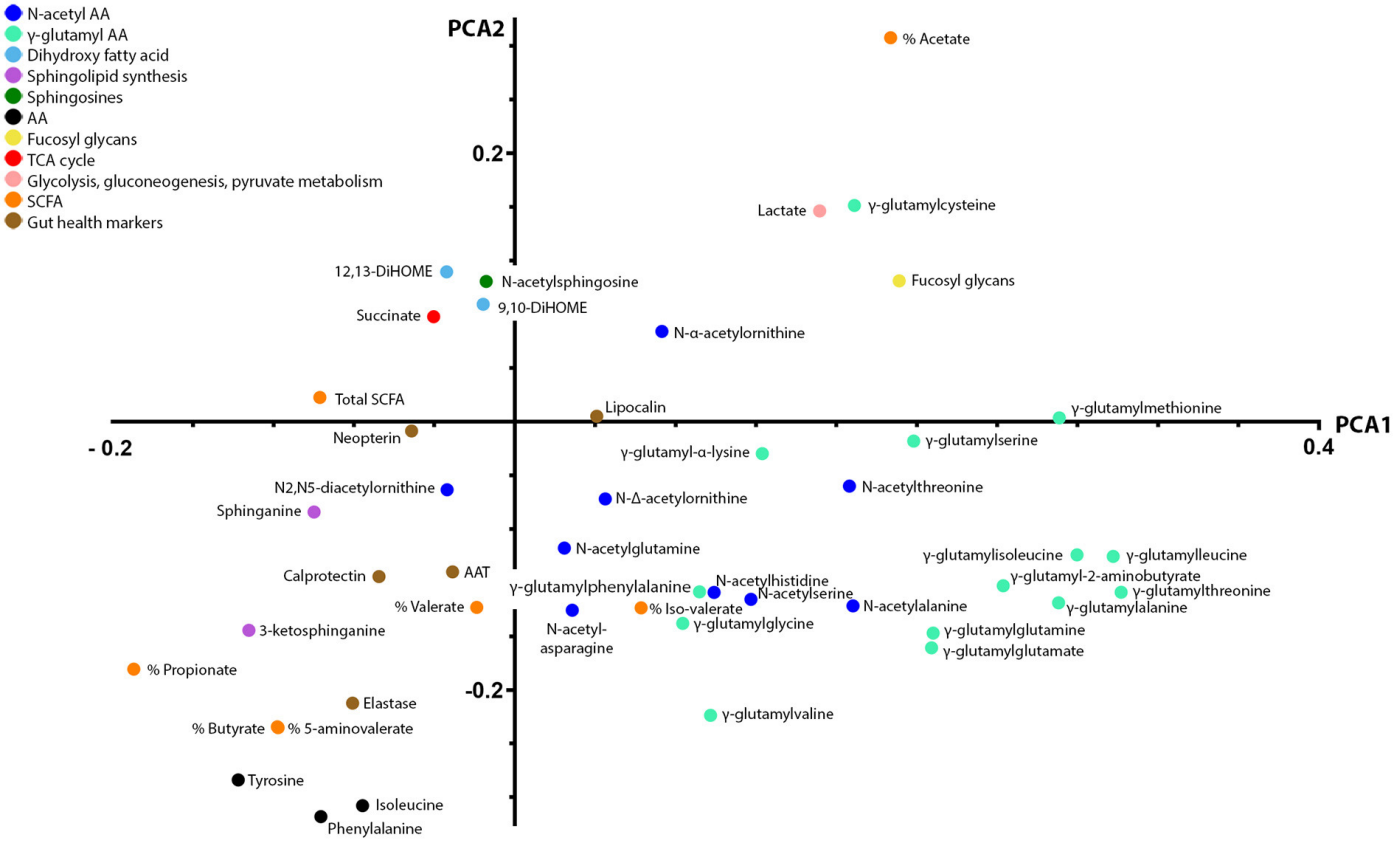
Principal component analysis loading plot displaying co-variations between ^1^H NMR and LC-MS stool metabolites and gut health markers. Principal component analysis was employed to model the main source of variances in the dataset and describe variable co-variations. The first two principal components accounted for 18 and 11% of the total variance in the combined data. In the principal component loading plot, each coordinate represents a single metabolite and describes each variable weight in a principal component. The coordinates of each variable are also providing information on how they correlate with one another. Along the first principal component, a positive association between the concentrations in stool calprotectin and elastase and the stool metabolites sphinganine, 3-keto-sphinganine, 5-aminovalerate, butyrate, and propionate was found. In addition, stool calprotectin and elastase showed a negative association with the stool metabolites acetate, lactate, fucosyl-glycans, and most gamma-glutamylated amino acids.

We explored the functional role of stool metabolites further by integrating them with the previously measured ^1^H NMR metabolites and gut health markers using unsupervised multivariate analysis. PCA was employed to model the main source of variances in the dataset and describe variable co-variations. Three principal components were calculated for the cross-validated PCA model to maximize the explained variance (R^2^X = 0.37). The first two principal components accounted for 18 and 11% of the total variance in the combined data. Data were visualized by means of principal loadings, where each coordinate represents a single metabolite or marker variable (Figure 3). Along the first principal component, a positive association between the concentrations in stool calprotectin and elastase and the stool metabolites sphinganine, 3-keto-sphinganine, 5-aminovalerate, butyrate, and propionate was found. In addition, stool calprotectin and elastase showed a negative association with the stool metabolites acetate, lactate, fucosyl-glycans, and most GGAAs (Figure 3).

## Discussion

**Figure 4:**
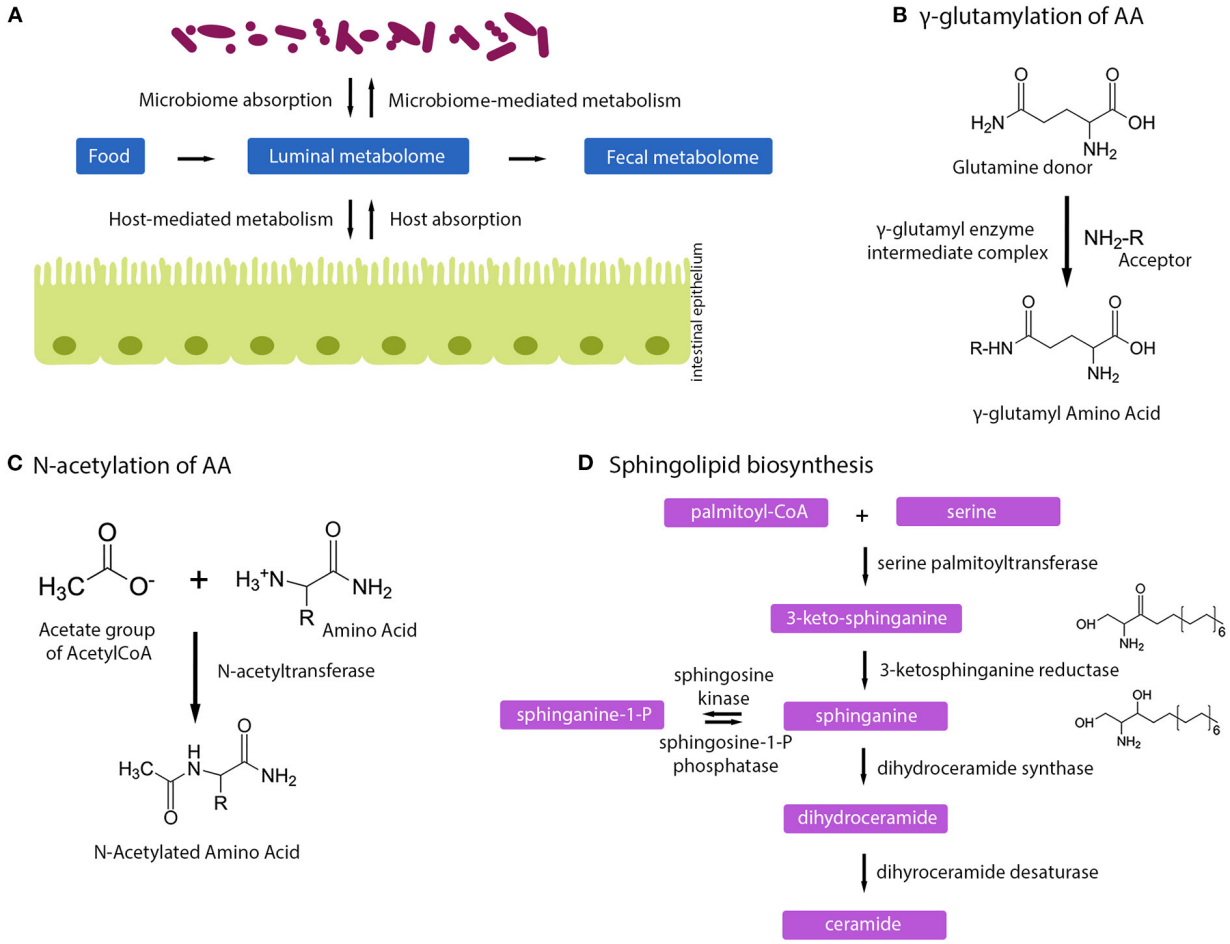
Human milk oligosaccharides influence host–microbe metabolome. **(A)** Schematic of how microbiome, host, and fecal metabolome can impact each other. **(B)** Gamma-glutamylation of amino acids happens *via* a gamma-glutamyl intermediate complex. **(C)** Mechanism of *N*-acetylation of amino acids. **(D)** Sphingolipid biosynthesis pathway.

In this study, we further elucidated the molecular basis for the earlier reported protective effect of 2-HMO formula against LRTIs over the course of the first year of life (17). We observed metabolic pathways that were modulated by HMOs and their relation to the observed lower incidence of LRTIs. Three molecular pathways correlated to HMO intake by infants: *N*-acetylation and gamma-glutamylation of amino acids, and (sphingo-) lipid metabolism (Figure 4). The latter two were also found to be prospectively correlated with LRTI incidence. The concentration of NAAs was found to be negatively associated with LRTIs, albeit to a lesser extent. These findings further corroborate our earlier report that highlighted the importance of HMO-stimulated *Bifidobacterium* species, specialized in HMO fermentation, thus contributing to a beneficial gut ecology (21).

The intake of HMOs has a profound effect on amino acid metabolism, illustrated by increased concentrations in NAAs and GGAAs, and reduced free amino acid content. Such a metabolic signature is indicative of a shift in bacterial *N*-acetylation and gamma-glutamylation of amino acids during protein and amino acid fermentation, with by-products being free NAAs and GGAAs available for absorption by the infant (Figures 4B,C). HMO-induced changes in the metabolic fluxes of the pentose phosphate pathway may further contribute to an excess of *N*-acetyl-CoA molecules being metabolized toward *N*-acetylation of amino acids. Upon intestinal absorption, both NAAs and GGAAs can then be taken up by other organs (e.g., liver) and either be hydrolyzed to acetate and a free AA by amino-acylases (28); or to glutamine and a free AA by glutamyltranspeptidases (28, 29).

Information on bacterial metabolism of NAAs is greatly lacking, except for *N*-acetyl-cysteine and glutathione metabolism (30, 31). While *N*-acetyl transferase activity may be limited in bacterial species, recent studies identified NAAs as a tissue-specific biomarker for respiratory tract infections in infants hospitalized with bronchiolitis (32). Children with rhinovirus, compared to respiratory syncytial virus, infections exhibited a higher concentration of NAAs in their nasopharyngeal metabolome with different NAAs showing different relations to the main microbial taxa present (32). This suggests that the changed NAA profile may be due to viral and microbial activities (32). It is therefore of interest to explore further the potential health implications of NAAs, including the potential role of an HMO-related increase in NAAs that may contribute to early-life immune development and protection later in life, as indicated by our results. Specific NAAs, like *N*-acetyl-carnosine, may confer antioxidant activity to the developing gut mucosa, thereby contributing to immune homeostasis (33, 34). In line with the numerous acetylated metabolites, we have recently reported the potential role of bifidobacterial-produced acetate as a contributor to the HMO-associated protection against LRTI in early life (21). Upon hydrolysis in the liver, the 1.5- to 2-fold increases in NAAs may subsequently be an important contributor to the circulating pool of acetate and further contributes to maintain its systemic immune protective effect.

We also found a strong and inverse correlation between GGAAs and LRTIs. HMOs were particularly associated with an increased content in gamma-glutamyl-2-aminobutyrate, gamma-glutamylalanine, gamma-glutamylcysteine, gamma-glutamylglutamate, gamma-glutamylisoleucine, gamma-glutamylleucine, gamma-glutamylmethionine, gamma-glutamylserine, gamma-glutamylthreonine, and gamma-glutamylcysteine. GGAAs are known to mediate various anti-inflammatory and antimicrobial effects (35), gamma-glutamylcysteine being particularly known to suppress TNF-α signaling and inflammatory cytokine expression in colonic intestinal epithelial cell cultures (36). GGAAs may thus contribute to some of the observed HMO health-promoting effects. Gamma-glutamyl-transpeptidase/transferase (GGT) enzymes catalyze the formation of GGAAs by transferring the gamma-glutamyl moieties from one to other amino acids or peptides; however, the same enzyme also has hydrolytic activity (37, 38). This impacts the metabolic fate of the molecules, and may further mediate different health-promoting effects, including immune protection and protection against oxidative stress (glutathione metabolism) (38, 39). Our integration of metabolite and microbiome data revealed that GGAAs were mostly correlated with *Bifidobacterium* species, namely, *B. bifidum, B. longum* subsp. *longum*, and *B. longum* subsp. *infantis* (Figure 2), thus indicating that these might harbor related metabolic pathways or affect others that drive GGAA metabolism. As HMO-stimulated *Bifidobacterium* species are central to mediate the protection from LRTIs (21), this novel observation signposts an additional mode of action. It is worth noting that gamma-glutamylation increases the stability of amino acids in the bloodstream, which can subsequently increase their bioavailability for metabolic functions in various tissues and organs (38). In *Helicobacter pylori*, the GGT gene and activity were shown to be important in the programming of dendritic cells toward a tolerogenic phenotype (40). Although the relevant molecular mechanism is not established, *H. pylori* GGT-induced immune tolerance may contribute to the protection from asthma observed in a mouse model (41).

It is worth noting that gamma-glutamylation was also found to contribute to increased absorption and bioavailability of glutamine for various gut and systemic functions, including optimal lymphocyte proliferation and production of cytokines by lymphocytes and macrophages (42). Dipeptides such as GGAAs are potentially better absorbed than free amino acids, as shown for glycyl-dipeptides, which appears to be the result of uptake by a system that has a greater transport capacity than amino acid carrier systems, thus minimizing competition among its substrates (43). Taken together, our observations indicate a role of HMOs in modulating luminal bacterial GGT-related metabolites, which subsequently may result in greater absorption and circulation in blood flow of amino acids and may contribute through yet-to-be-discovered mechanisms, resulting in a lower risk to experience LRTIs in infancy.

Our analysis further revealed specific changes in lipid metabolism, especially for sphingolipids and inflammatory signaling fatty acids, which were reduced in the stool of infants fed with a 2-HMO formula compared to the control feeding group. The observed lower amounts may reflect differential formation of bioactives by the gut–microbial co-metabolism or differential absorption of those bioactives in 2-HMO and control formula-fed infants. The former possibility may imply age-specific effects of those bioactive lipids, which needs to be further explored. These observations are novel and of importance in a field where sphingolipids are more and more seen as potent bioactives with a role in innate immunity, protection against pathogen invasion and bacterial infection (44). Sphingolipids are a lipid class characterized by a long-chain amino alcohol backbone, with a critical role in structural and signaling functions in eukaryotes (45). They are abundant in the microvillar membrane of intestinal epithelial cells, where they are essential for structural integrity and may act as receptors for toxins, viruses, and bacteria (46). They act as bioactive lipid messengers, influencing numerous cellular functions, including growth, differentiation, apoptosis of both epithelial and immunocompetent cells in the gastrointestinal tract, and the progress of inflammation and responsiveness of the mucosal cells to pathogens (46–48).

Our integration of metabolite and microbiome data found that major intermediates in sphingolipid biosynthesis, sphinganine, and 3-keto-sphinganine, reduced in infants fed the 2-HMO formula, were positively correlated with *Bacteroides* species (Figures 2, 4D). Sphingolipid-producing bacteria include many species of the Bacteroidota phylum (i.e., *Bacteroides, Parabacteroides, Prevotella*, and *Prophyromonas*) (49). As mentioned, the observed lower concentrations of such molecules in the stools of infants fed with a 2-HMO formula may either suggest greater absorption or a reduction in gut microbial formation. Sphingolipids produced by gut bacteria were shown to enter host metabolic pathways impacting sphingolipid levels (50). Hence, changes observed in stool may be a proxy for changes in systemic levels, where sphingolipids may contribute to HMO-mediated benefits through immune-mediated signaling.

A role of bacterial sphingolipids in immune system maturation has been described earlier (45). Recently, Lee-Sarwar et al. (51) also reported how microbial-associated sphingolipids, especially metabolites in the *de novo* sphingolipid synthesis pathway, may be linked with protection from food allergy. Here, we described that gut health markers calprotectin and elastase were positively associated with the stool concentration of sphinganine and 3-keto-sphinganine, which further supports a potential role in maintaining intestinal homeostasis and inflammation early in life. A recent study has highlighted the interactions between the common gut commensal *B. fragilis* and the host, describing how the specific sphingolipid alpha-galactosylceramide may directly modulate host intestinal natural killer T cells (52). Other investigations describe the importance of sphinganine in relation to skin barrier function and atopic dermatitis severity (53). Therefore, both HMO-modulated *Bifidobacterium* and *Bacteroides* species may be of importance to mediate early-life protection from LRTIs (21).

Based on previously identified dihydroxylated fatty acids (DiHOME) in relation to immunity (54), we specifically looked for those in our data. HMO-driven differences in lipid metabolism are marked by the reduced abundance of two DiHOME species, which could be indicators of an altered inflammatory or immune response (55, 56). It is worth noting that DiHOMEs were earlier shown to modulate the relative abundance of allergy-protective regulatory T cells (54, 57). The fatty acid 12,13-DiHOME was shown to be bacterial derived and play a role in the acute response to inflammation (57) and may suppress regulatory T cell function by regulating PPARgamma activity (58).

Taken together, our analysis generated novel insights into how HMO feeding impacts the developing gut microbiome–host metabolome in early infancy, and how this relates to the lower incidence of LRTIs observed in infants fed a 2-HMO formula, compared to those fed a control formula. However, we want to highlight some study limitations. We based our analysis on home-collected fecal samples. Although we instructed parents to collect within 48 h of the 3-month study visit, freeze the fecal sample in the home freezer, and bring in the sample frozen in a cooling box (that was provided), we cannot exclude variability introduced by home collection. Due to the randomization, this should however not have affected the group comparison. Furthermore, we based our interpretation on the metabolites found in feces. Inherent to such an approach, we cannot know whether higher amounts of certain metabolites reflect lower absorption by the infant gut or higher production by the gut microbiome. However, our analysis showcases the impact of HMOs on amino acid and lipid metabolism and generates new hypotheses on how these metabolic pathways might impact the gut–lung axis.

## Data availability statement

The data supporting the findings of this study are available within the article and its Supplementary materials. Metabolomics analysis data plus descriptor, plus gut health markers, and all analysis data used in the integration of MGSs and metabolomics data are available in the Supplementary Files. Metagenomic sequencing data were submitted to the European Nucleotide Archive under the accession number PRJEB54707 (https://www.ebi.ac.uk/ena/browser/view/PRJEB54707).

## Ethics statement

The studies involving human participants were reviewed and approved by Ethische Toetsingscommissie, Jessa Ziehenhuis. Hasselt, Belgium B243201216048 and Ethics Committee of Azienda Ospedaliera Universitaria Policlinico Paolo Giaccone di Palermo, verbale No 05/2012. Written informed consent to participate in this study was provided by the participants' legal guardian/next of kin.

## Author contributions

F-PM and NS designed the study. BB oversaw metagenomics analysis. DM carried out sequencing. HK and AE performed integration of metabolomics and metagenomics data. F-PM analyzed and interpreted the metabolomics data. F-PM, NS, and HT analyzed all data and drafted the manuscript and figures. All authors read and approved the final version of the manuscript.

## Funding

This study received funding from Nestlé Nutrition, Société des Produits Nestlé S.A., Switzerland. The funder was not involved in the study design, collection, analysis, interpretation of data, the writing of this article or the decision to submit it for publication.

## Conflict of interest

Authors HT, BB, NS, F-PM, and DM were employees of Société des Produits Nestlé S.A., Switzerland at the time of the study. HK and AE were employees of Clinical Microbiomics, Denmark.

## Publisher's note

All claims expressed in this article are solely those of the authors and do not necessarily represent those of their affiliated organizations, or those of the publisher, the editors and the reviewers. Any product that may be evaluated in this article, or claim that may be made by its manufacturer, is not guaranteed or endorsed by the publisher.
