# Supplementary material for: Host–microbial co-metabolites modulated by human milk oligosaccharides relate to reduced risk of respiratory tract infections
Source: Front Nutr. 2022 Aug 4;9:935711. doi: 10.3389/fnut.2022.935711 (PMC9386273; doi:10.3389/fnut.2022.935711)
Supplement: Supplementary file 1 [file Data_Sheet_1.docx]

Supplementary Material

# Supplementary methods

## Clinical trial

The trial was designed as a randomized, controlled, multicenter clinical trial and is registered with number NCT01715246 at www.clinicaltrial.gov. Extensive details of the clinical trial were previously described for the full trial population in Puccio *et al*. (1), including randomization and blinding procedures, and for the per protocol formula-fed trial population by Berger *et al*.(3). A random subset of 80 infants from the latter population was further studied here (Fig. S1).

Primary objective of the trial was to evaluate the non-inferiority of weight gain from enrolment to 4 months of age, when comparing infants fed a control formula and a test formula, the latter which was supplemented with 2 HMOs, i.e. 2’Fucosyllactose (2’FL) and Lacto-N-neotetraose (LNnT) (Fig. S1). A plethora of secondary objectives was also studied, including evaluation of differences between the two groups concerning body weight, length, digestive tolerance, stool microbiome, etc. Full details and outcomes for these objectives are described in aforementioned papers(1, 3).

Healthy, full-term male and female infants of 0 to 14 days of age were enrolled in the formula-fed arms of the trial, if they were exclusively formula-fed at time of enrollment. Randomized assignment of subjects to the 2-HMO or Control arm of the trial was ensured by using a permuted block algorithm with Medidata Balance (New York, USA), using mode of delivery (vaginal vs. C-section) and gender as stratification factors. Up till 4 months of age, the enrolled infants exclusively received either 2-HMO or Control formula, according to their weight, age and appetite. Upon weaning, introduction of complementary food was allowed, combined with continued feeding with Control and 2-HMO formula through 6 months of age. After this, both groups received the same intact protein cow’s milk-based follow-up formula for feedings from 6 to 12 months of age.

## Reporting of adverse events

As part of the clinical trial reporting, adverse events were recorded, as described by Puccio *et al.*(1)*.* In short, adverse events were logged, coded and categorized by a single physician (not involved in conducting the study), using the Medical Dictionary for Regulatory Activities (MedDRA) System, Organ, and Class (SOC) categories as well as Preferred Terms (PTs) within each SOC category.

In this work, we focus on the reported Lower Respiratory Tract Infections (LRTIs) adverse event cluster. This cluster includes PTs as: Bronchiolitis, Bronchitis, Pneumonia, LRTI, LRTI viral, RSV (respiratory syncytial virus) bronchiolitis, RSV bronchitis, respiratory infection viral. A subset of infants that experienced between 0 and multiple LRTIs over the course of their first year of life, were included in this study (Fig. S1). In case an infant only experienced an LRTI during their first 3 months of life, it was excluded from our analysis. An infant was thus considered as LRTI positive if it experienced at least one LRTI between 3 and 12 months of age. This resulted in a total of 28 infants experiencing LRTIs and 52 that did not have any reported LRTIs over the course of their first year of life (Fig. S1).

## Stool collection

Stool samples were collected at home by the parents of the subjects, within 48 hours preceding the 3-month visit. Parents were supplied with a kit (insulated bag, ice pack, spatula pots, sealable plastic bags and an instruction sheet) and asked to collect two samples. Fecal samples were to be stored at home in a -20°C freezer, prior to transport in the insulated bag with the frozen ice pack to the visit site and further storage at -80°C. Samples were then shipped to the analysis center on dry ice and kept frozen at -80°C until analysis.

## Stool gut health markers

Gut health markers were analyzed as described earlier (2). In short, calprotectin, α1-antitrypsin and elastase were quantified by Biotecon Diagnostics GmbH (Germany). More in particular, quantification of α1-antitrypsin was performed using a stool extraction kit and Enzyme-Linked Immuno-Sorbent Assay (ELISA) provided by Immundiagnostik AG (Germany). Elastase was quantified using the ScheBo® Quick-PrepTM E1-stool extraction system (ScheBo Biotech, Germany) and the ScheBo® Pancreatic Elastase 1 assay (ScheBo® Biotech). Calprotectin was extracted and quantified with the Bühlmann Smart-Prep (Germany) stool extraction system with the EK-CAL ELISA kit (Bühlmann).

## Microbiome profiling

**Fecal DNA extraction and sequencing.** Total DNA was extracted using the QIAamp DNA Stool Mini Kit (Qiagen), following manufacturer's instructions, with the addition of a series of mechanical disruption steps (4 × 60 s) using a FastPrep apparatus and Lysing Matrix B tubes (MP Biochemicals) (4). DNA libraries for metagenomic sequencing were prepared using the Nextera™ XT DNA Sample Preparation Kit (Illumina), and sequenced on an Illumina HiSeq instrument with paired-end 2×100 bp reads, using six high output Flow Cells.

**Sequencing data processing.** Quality control of the resulting FASTQ sequencing read files was performed using KneadData (v.0.6.1) to remove low-quality bases and reads derived from the host genome. Using Trimmomatic (v.0.36), reads were quality trimmed by removing Nextera™ adapters, leading and trailing bases with a Phred score below 20 and trailing bases in which the Phred score over a window of size 4 dropped below 20. Trimmed reads shorter than 90 bases were discarded. Reads mapping to the human reference genome GRCh38 were discarded (Bowtie2 v.0.2.3.2 (5) at default settings). Read pairs in which both reads passed filtering were retained and classified as high-quality non-host (HQNH) reads.

**Gene catalog and metagenomic species (MGS) definitions.** The Clinical Microbiomics in-house Human Gut 22M gene catalog (containing 22,459,186 ‬microbial genes) was used as a reference gene catalog and the corresponding set of 1,273 MGS definitions for MGS abundance profiling. The MGSs were built based on >5000 deep-sequenced human adult and infant gut samples using an approach based on the metagenomic species concept (6) and have highly coherent abundance and base composition in a set of 1776 independent reference human gut samples.

**Mapping reads to gene catalog.** Trimmed, human-filtered reads were mapped to the gene catalog using BWA mem (v. 0.7.16a) (7). PCR duplicates were removed using Samtools (v.1.6). An individual read was considered mapped to a gene if the mapping quality (MAPQ) was ≥ 20 and the read aligned with ≥ 95 % identity over ≥ 90 bp. However, if > 10 bases of the read did not align to the gene or extend beyond the gene, the read was considered unmapped. Reads meeting these criteria except for the MAPQ threshold were considered multi-mapped. Each read pair was counted as either I) mapped to a specific gene, if both individual reads mapped to the same gene, or one read mapped to a gene and the other was unmapped, multimapped, or mapped to another gene in the same MGS, or II) multi-mapped, if both reads were multimapped, or mapped to genes in different MGSs, or if one read was multimapped and the other one unmapped, or III) unmapped, if neither individual read mapped. The resulting gene count table, of number of mapped read pairs for each gene, was used to calculate the relative abundance of each MGS.

**Taxonomical annotation of MGSs.** To taxonomically annotate an MGS, we blasted its genes against the NCBI RefSeq genome database (2018-03-23) and used rank-specific annotation criteria. Specifically, we assigned a taxon to an MGS if at least M % of its genes were mapped to the taxon and no more than D % of its genes were mapped to a different taxon. We only considered blast hits with an alignment length ≥ 100 bp, ≥ 50 % query coverage and % identity ≥ PID. Here we define: PID = (95, 95, 85, 75, 65, 55, 50, 45); M = (75, 75, 60, 50, 40, 30, 25, 20); and D = (10, 10, 10, 20, 20, 20, 20, 15) for subspecies, species, genus, family, order, class, phylum, and superkingdom, respectively. Finally, we processed each MGS with CheckM (8), and updated our annotation with the CheckM result if this resulted in a lower taxonomic rank.

**MGS relative abundance calculation**. For each MGS, a signature gene set had been previously defined, as the 100 genes optimized for accurate abundance profiling of the MGS. An MGS count table was created by counting the number of reads mapped to the MGS signature genes per sample. An MGS was considered detected if reads from a sample mapped to at least three of its signature genes; measurements that did not satisfy this criterion were set to zero. The MGS count table was normalized according to effective gene length and then normalized sample-wise to sum to 100 %, resulting in relative abundance estimates of each MGS.

## Metabolomics

**Proton Nuclear Magnetic Resonance Spectroscopy Metabolomics.** The stool biochemical composition was analyzed using a metabolomic approach based on proton Nuclear Magnetic Resonance Spectroscopy (^1^H NMR) and a previously published method (9, 10). Briefly, 80-100mg of frozen stool was sampled from the stool collection tube, weighed and freeze-dried. Dried samples were suspended in 1.2 mL of deuterated phosphate buffer solution 0.2 M KH2PO4, containing 0.3 mM of sodium azide as antibacterial agent and 1 mM of sodium 3-(trimethylsilyl)-[2,2,3,3-2H4]-1-propionate as ^1^H NMR chemical shift reference. The homogenates were centrifuged at 17,000 x *g* for 10 minutes and 5500 µL of the supernatant was transferred into 5 mm NMR tubes. ^1^H NMR metabolic profiles were acquired with a Bruker Avance III 600 MHz spectrometer equipped with a 5 mm cryoprobe at 300K (Bruker, Biospin, Germany) using a standard pulse sequence and a spin-echo pulse sequence with water suppression and processed using TOPSPIN (vs 3.2., Bruker) software package. Data processing and analysis was conducted as previously reported (9, 10). Quantitative profiles of selected metabolites were acquired, including amino acids (Phenylalanine, Tyrosine, Isoleucine), short chain fatty acids (propionate, butyrate, acetate, valerate, isovalerate, 5-amino-valerate), organic acids (lactate, succinate) and carbohydrates (fucosyl-glycans), as recently reported (2).

**Mass Spectrometry metabolomics.** Complementary analysis using mass spectrometry-based metabolomics was conducted in collaboration with Metabolon Inc. (München, Germany).

**Sample preparation.** Briefly, samples were prepared using the automated MicroLab STAR® system from Hamilton Company. Samples were extracted with methanol under vigorous shaking for 2 min (Glen Mills GenoGrinder 2000) followed by centrifugation. The resulting extract was divided into ﬁve fractions: two for analysis by two separate reverse phase Ultrahigh Performance Liquid Chromatography-Tandem Mass Spectroscopy (RP/UPLC-MS/MS,) methods using positive ion mode electrospray ionization (ESI), one for analysis by RP/UPLC-MS/MS using negative ion mode ESI, one for analysis by HILIC/ UPLC-MS/MS using negative ion mode ESI, and one reserved for backup. Samples were placed brieﬂy on a TurboVap® (Zymark) to remove the organic solvent. The sample extracts were stored overnight under nitrogen before further preparation for analysis.

**Ultrahigh Performance Liquid Chromatography-Tandem Mass Spectroscopy (UPLC-MS/MS).** All methods utilized a Waters ACQUITY ultra-performance liquid chromatography (UPLC) and a Thermo Scientific Q-Exactive high resolution/accurate mass spectrometer interfaced with a heated electrospray ionization (HESI-II) source and Orbitrap mass analyzer operated at 35,000 mass resolution. The sample extract was dried and then reconstituted in solvents compatible with each of the four methods. Each reconstitution solvent contains a series of standards at fixed concentrations to ensure injection and chromatographic consistency. One aliquot was analyzed using acidic positive ion conditions, chromatographically optimized for more hydrophilic compounds. In this method, the extract was gradient-eluted from a C18 column (Waters UPLC BEH C18-2.1×100 mm, 1.7 µm) using water and methanol, containing 0.05% perfluoropentanoic acid (PFPA) and 0.1% formic acid (FA). A second aliquot was also analyzed using acidic positive ion conditions, but this time chromatographically optimized for more hydrophobic compounds. In this method, the extract was gradient eluted from the aforementioned C18 column using methanol, acetonitrile, water, 0.05% PFPA and 0.01% FA, and was operated at an overall higher organic content. A third aliquot was analyzed using basic negative ion optimized conditions, using a separate dedicated C18 column. The basic extracts were gradient-eluted from the column using methanol and water, however with 6.5mM Ammonium Bicarbonate at pH 8. The fourth aliquot was analyzed via negative ionization following elution from a HILIC column (Waters UPLC BEH Amide 2.1×150 mm, 1.7 µm) using a gradient consisting of water and acetonitrile with 10mM Ammonium Formate, pH 10.8. The MS analysis alternated between MS and data-dependent MSn scans using dynamic exclusion. The scan range varied slightly between methods, but covered approximately 70-1000 m/z.

**Quality control.** Several types of quality control samples were analyzed in concert with the experimental samples. These include: 1) technical replicate samples derived from a pool of well-characterized human plasma (MTRX) or, alternatively, generated by combining a small portion of each (non-plasma) experimental sample (CMTRX), spaced evenly among experimental samples; 2) extracted water samples (process blanks) and solvent blanks; and 3) a cocktail of QC standards, carefully chosen not to interfere with the measurement of endogenous compounds, spiked into every analyzed sample, allowing instrument performance monitoring and aiding with chromatographic alignment. Instrument variability was determined by calculating the median relative standard deviation (RSD) for the standards that were added to each sample prior to injection into the mass spectrometers. Overall process variability was determined by calculating the median RSD for all endogenous metabolites (i.e., non-instrument standards) present in each of the pooled MTRX (or CMTRX) technical replicate samples. Experimental samples were randomized across the platform run, with QC samples spaced evenly among injections.

**Bioinformatics.** Analysis was performed using an informatics system consisting of four major components, the Laboratory Information Management System (LIMS), the data extraction and peak-identiﬁcation software, data processing tools for QC and compound identiﬁcation. The hardware and software foundations for these informatics components are the LAN backbone and database servers running Oracle 10.2.0.1 Enterprise Edition.

The purpose of the Metabolon LIMS system is to enable fully auditable laboratory automation through a secure, easy-to-use, highly specialized system. The scope of the Metabolon LIMS system encompasses sample accessioning, sample preparation, instrumental analysis and reporting, and advanced data analysis. All the subsequent software systems are grounded in the LIMS data structures, which was modified to leverage and interface the in-house information extraction and data visualization systems, as well as third party instrumentation and data analysis software.

Raw data were extracted, peak-identiﬁed, and QC processed using hardware and software from Metabolon Inc. (München, Germany), built on a web service platform (Microsoft’s.NETtechnologies). Metabolon maintains a library based on authenticated standards that contains the retention time/index (RI), mass to charge ratio (m/z), and chromatographic data (including MS/MS spectral data) of all molecules present in the library. Biochemical identiﬁcations were based on the retention index within a narrow RI window of the proposed identiﬁcation; accurate mass match to the library ±10 ppm; and the MS/MS forward and reverse scores. All three data points were utilized to distinguish and differentiate potentially similar biochemicals. More than 4500 commercially available puriﬁed standard compounds have been acquired and registered into LIMS to facilitate analysis on all platforms. Additional mass spectral entries have been created for structurally unnamed biochemicals, which have been identiﬁed by virtue of their recurrent nature.

Peaks were quantiﬁed as area-under-the-curve detector ion counts. For studies spanning multiple days, we performed a data adjustment step to correct block variation resulting from instrument inter-day tuning differences. Such adjustments were conducted by registering the daily medians to equal one and adjusting each data point proportionately (block correction).

A variety of curation procedures were performed to ensure that a high-quality data set was available for statistical analysis and data interpretation. The QC and curation processes are designed to ensure accurate and consistent identification of true chemical entities, and to remove those representing system artifacts, mis-assignments, redundancy, and background noise. Metabolon data analysts use internally developed visualization and interpretation software to confirm the consistency of peak identification among the various samples. Library matches for each compound are checked for each sample and corrected if necessary.

# Supplementary Data File

Full overview of metabolites in stool content of infants fed either control or 2-HMO formula, with fold change and associations to LRTIs. Interdomain correlation between metagenomic species (MGS) and metabolite abundances and association with 2-HMO formula and LRTIs. The sheet ‘Interdomain correlation’ shows all 6,200 pairwise correlations between MGSs and metabolites. The sheets ‘Metabolites’ and ‘MGSs’ show the association with 2-HMO formula and LRTIs for the subset of metabolites and MGSs with at least one significant correlation (FDR ≤ 10%), respectively, including median and mean concentration/relative abundance and prevalence for each group.

# Supplementary Figures and Tables

## Supplementary table

**Supplementary Table 1. Stool biomarkers at 3 months of age.**

| Stool biomarkers | Control formula | 2-HMO  formula | Control formula with LRTIs | 2-HMO formula with LRTIs | Control formula without LRTIs | 2-HMO formula without LRTIs |
| --- | --- | --- | --- | --- | --- | --- |
| Calprotectin [µg/gFW] | 576  ±571.6 | 399.2  ±420.3 | 739.4  ±646.5 | 456.3  ±352.6 | 453.5  ±472.6 | 378.8  ±440.1 |
| α-antitrypsin [µg/gFW] | 217.2  ±191.8 | 229.6  ±186.2 | 203.4  ±133.2 | 191.1  ±140.8 | 227.5  ±225.5 | 243.4  ±198.2 |
| Elastase [µg/gFW] | 2765.1  ±1071.3 | 2674.4  ±974.8 | 2880.5  ±898.1 | 2357.8  ±976 | 2678.5  ±1177.3 | 2787.6  ±949.1 |
| Lipocalin [ng/gFW] | 8.4 ±3 | 8.1 ±4.1 | 8.1 ±2.6 | 9.1 ±4.9 | 8.6 ±3.3 | 7.8 ±3.6 |
| Neopterin [ng/gFW] | 6.1 ±5.6 | 5.2 ±2.3 | 5.2 ±1.7 | 5.4 ±1.3 | 6.8 ±7.2 | 5.1 ±2.6 |

gFW = gram fecal weight

## Supplementary Figure

**
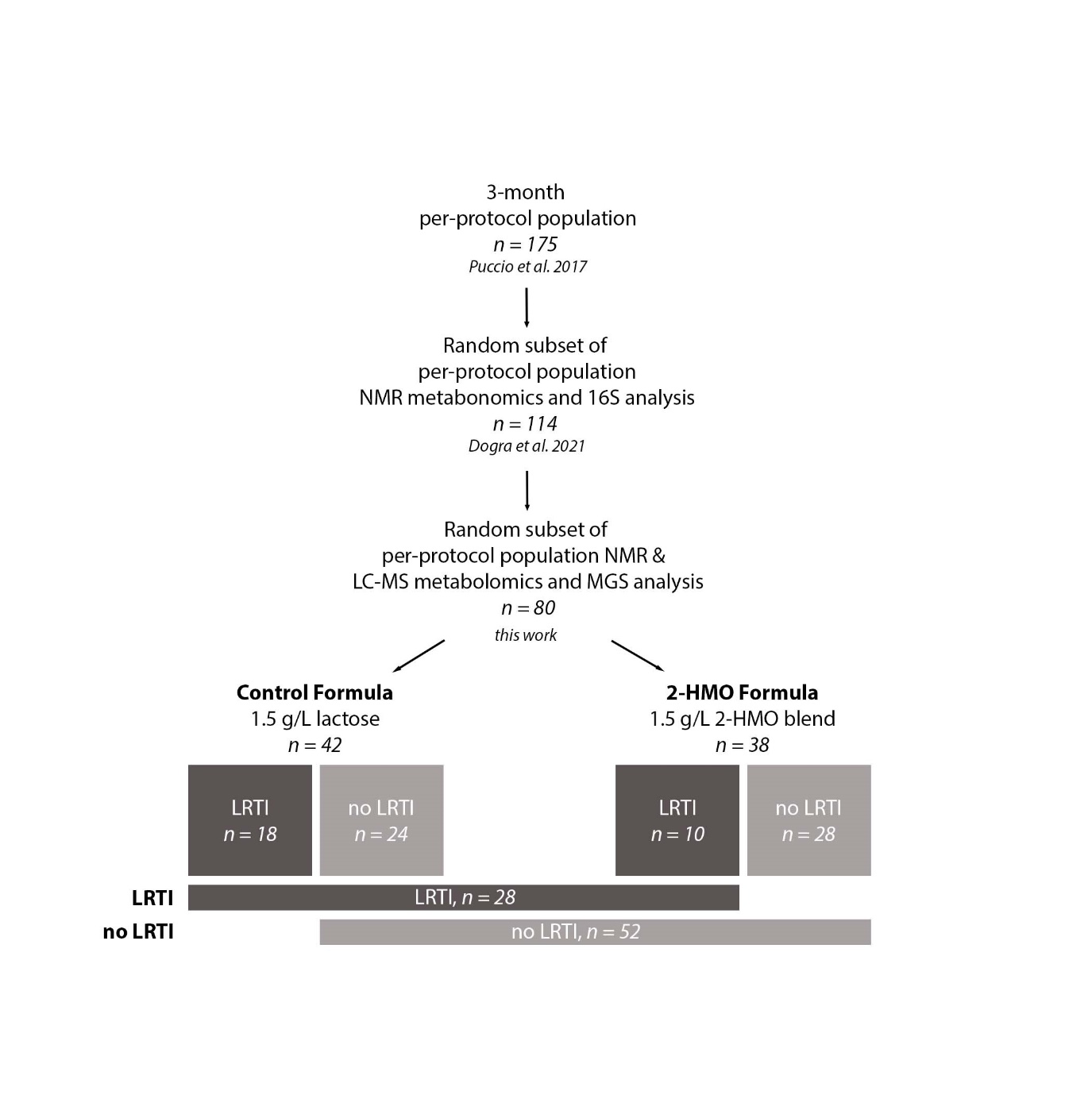
**

**Supplementary Figure 1. Overview of randomized clinical trial samples utilized in this study.**

Infants were randomly distributed over a control and 2-HMO formula-fed arm. At the three months timepoint, 175 infants were assessed as being per-protocol(1). A random subset of fecal samples of 114 infants was selected for NMR metabolomics and 16S analysis(2), whilst in the current paper a further subset of 80 infant fecal samples was further subjected to metagenomics and metabolomics analyses. The selection of the 80 samples was random, and mostly based on the availability of enough fecal matter to perform further analyses. This resulted in 42 infants being fed control formula and 38 2-HMO formula. A total of 28 experienced LRTIs between their third and twelfth month of life.

# References

# 1. Puccio G, Alliet P, Cajozzo C, Janssens E, Corsello G, Sprenger N, et al. Effects of Infant Formula With Human Milk Oligosaccharides on Growth and Morbidity: A Randomized Multicenter Trial. J Pediatr Gastroenterol Nutr. 2017;64(4):624-31.

# 2. Dogra SK, Martin FP, Donnicola D, Julita M, Berger B, Sprenger N. Human Milk Oligosaccharide-Stimulated Bifidobacterium Species Contribute to Prevent Later Respiratory Tract Infections. Microorganisms. 2021;9(9).

# 3. Berger B, Porta N, Foata F, Grathwohl D, Delley M, Moine D, et al. Linking Human Milk Oligosaccharides, Infant Fecal Community Types, and Later Risk To Require Antibiotics. mBio. 2020;11(2).

# 4. Junick J, Blaut M. Quantification of human fecal bifidobacterium species by use of quantitative real-time PCR analysis targeting the groEL gene. Appl Environ Microbiol. 2012;78(8):2613-22.

# 5. Langmead B, Salzberg SL. Fast gapped-read alignment with Bowtie 2. Nat Methods. 2012;9(4):357-9.

# 6. Nielsen HB, Almeida M, Juncker AS, Rasmussen S, Li J, Sunagawa S, et al. Identification and assembly of genomes and genetic elements in complex metagenomic samples without using reference genomes. Nat Biotechnol. 2014;32(8):822-8.

# 7. Li H, Durbin R. Fast and accurate short read alignment with Burrows-Wheeler transform. Bioinformatics. 2009;25(14):1754-60.

# 8. Parks DH, Imelfort M, Skennerton CT, Hugenholtz P, Tyson GW. CheckM: assessing the quality of microbial genomes recovered from isolates, single cells, and metagenomes. Genome Res. 2015;25(7):1043-55.

# 9. Martin FP, Moco S, Montoliu I, Collino S, Da Silva L, Rezzi S, et al. Impact of breast-feeding and high- and low-protein formula on the metabolism and growth of infants from overweight and obese mothers. Pediatr Res. 2014;75(4):535-43.

# 10. Moco S, Collino S, Rezzi S, Martin FP. Metabolomics perspectives in pediatric research. Pediatr Res. 2013;73(4 Pt 2):570-6.
